# The PDZ-Ligand and Src-Homology Type 3 Domains of Epidemic Avian Influenza Virus NS1 Protein Modulate Human Src Kinase Activity during Viral Infection

**DOI:** 10.1371/journal.pone.0027789

**Published:** 2011-11-14

**Authors:** Laura Bavagnoli, William G. Dundon, Anna Garbelli, Bianca Zecchin, Adelaide Milani, Geetha Parakkal, Fausto Baldanti, Stefania Paolucci, Romain Volmer, Yizeng Tu, Chuanyue Wu, Ilaria Capua, Giovanni Maga

**Affiliations:** 1 Institute of Molecular Genetics National Research Council, Pavia, Italy; 2 World Organization for Animal Health, Food and Agriculture Organization and National Reference Laboratory for Newcastle Disease and Avian Influenza, Istituto Zooprofilattico Sperimentale delle Venezie, Legnaro, Italy; 3 Molecular Virology Unit, Virology and Microbiology, Fondazione Istituto Ricovero e Cura a Carattere Scientifico Policlinico S. Matteo, Pavia, Italy; 4 Université de Toulouse, Institut National Polytechnique, Ecole Nationale de Veterinaire, Unitè Mixte de Recherche 1225, Interactions Hotes-Agents Pathogènes, Toulouse, France; 5 Department of Pathology, University of Pittsburgh, Pittsburgh, Pennsylvania, United States of America; Johns Hopkins University - Bloomberg School of Public Health, United States of America

## Abstract

The Non-structural 1 (NS1) protein of avian influenza (AI) viruses is important for pathogenicity. Here, we identify a previously unrecognized tandem PDZ-ligand (TPL) domain in the extreme carboxy terminus of NS1 proteins from a subset of globally circulating AI viruses. By using protein arrays we have identified several human PDZ-cellular ligands of this novel domain, one of which is the RIL protein, a known regulator of the cellular tyrosine kinase Src. We found that the AI NS1 proteins bind and stimulate human Src tyrosine kinase, through their carboxy terminal Src homology type 3-binding (SHB) domain. The physical interaction between NS1 and Src and the ability of AI viruses to modulate the phosphorylation status of Src during the infection, were found to be influenced by the TPL arrangement. These results indicate the potential for novel host-pathogen interactions mediated by the TPL and SHB domains of AI NS1 protein.

## Introduction

**Figure 1:**
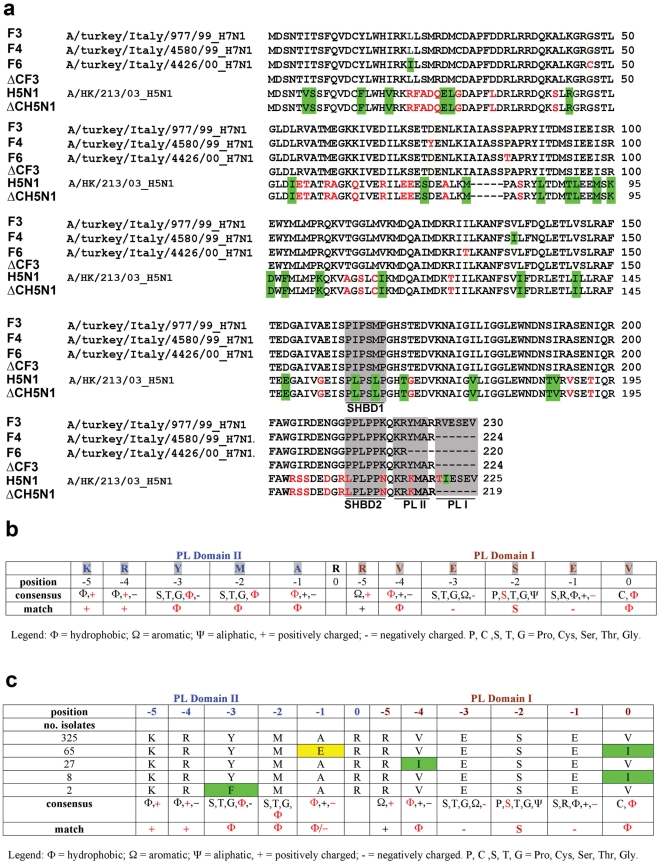
The NS1 protein of H7N1 avian influenza viruses has a new tandem PDZ-ligand domain. **a**. Sequence alignment of the NS1 proteins from Italian H7N1 AI isolates and the H5N1 NS1 protein. Equivalent aminoacids are shaded in green; non-equivalent aminoacid are in red. SHBD, Src-homology type 3 binding domains. PL, PDZ-ligand domain. Alignments were generated with the program ClustalW. **b**. Comparison of the amino acid specificities of PL domains I and II of the H7N1 NS1 protein with the consensus specificity map for cellular PL domains. Matching aminoacids are shown in red. **c**. Comparison of the amino acid specificities of PL domains I and II of the different AI virus NS1 protein with the consensus specificity map for cellular PL domains. Matching aminoacids are shown in red. Equivalent aminoacidic substitions with respect to the H7N1 NS1 protein are shaded in green. Non-equivalent substitutions are shaded in yellow.

Avian influenza (AI) viruses pose significant threats to both animal and human health. Approximately 200 million birds have died or have been culled worldwide as a result of AI. In addition, since 1997, AI viruses of the H5 or H7 subtype have crossed the species barrier and caused fatal disease in humans [1], [2], [3], [4]. These events, in addition to the emergence of the triple-reassortant of avian and swine genes H1N1v as a pandemic virus in 2009 [5], warn us of the potential generation of another human pandemic virus. Thus, understanding the pathogenic determinants of AI viruses and their modifications in the course of natural epidemics is important for the identification of natural reservoirs of potential pandemic viruses. The NS1 protein of AI viruses is an important pathogenicity determinant [6], [7], and was shown to possess a PDZ-ligand (PL) motif, enabling it to interact with a wide range of cellular proteins [8]–[11] and influencing pathogenicity [12]–[14]. One of the most severe AI outbreaks in European poultry was caused by an H7N1 subtype virus between 1999 and 2001. Longitudinal analysis of low pathogenic (LP) and high pathogenic (HP) AI H7N1 isolates circulating during the outbreak [15], revealed that the H7N1 LP viruses at the beginning of the epidemic had a full length NS1 protein (F3 protein, Fig. **1 a**), while the HP isolates circulating during the peak weeks showed a truncation of the last C-ter six amino acids (aa) of NS1 (F4_Δ225–230_ protein, Fig. **1 a**). At the end of the epidemic, the majority of circulating AI viruses were LP isolates, with a C-ter deletion of 10- aa in the NS1 protein (F6_Δ221–230_ protein, Fig. **1 a**). H5 and H7 AI viruses are the only subtypes causing high pathogenic infections in poultry and to possess the potential to be transmitted to humans. Thus, changes in the pathogenic potential of these AI viruses during extensive epidemics might represent a potential threat to human health. Since the observed truncations in H7N1 NS1 protein spanned the PDZ-ligand (PL) domain, which has been documented to influence pathogenicity through the interaction with host cell proteins, we investigated whether these truncations might alter the ability of the AI NS1 protein to interact with human host factors.

## Results

### Identification of a novel tandem PDZ-ligand domain in epidemic H7N1 virus

PL domains are specific sequences, usually located in the last 5 to 6- C-ter aa of cellular proteins, that recognize the structural protein-protein interaction module PDZ [16], [17]. A map of the amino acid specificity for PL domains has been derived starting from the physico-chemical characteristics of the aa present with higher frequency in the PL sequences recognized by different PDZ domains [18]. Based on this specificity map, we identified in the last 12- aa of the H7N1 NS1 proteins two partially overlapping PL motifs (indicated as PL domain I and II, respectively, in Fig. **1 a** and **b**). BLAST search using the consensus sequence for this novel tandem PL (TPL) domain as bait retrieved 427 avian influenza NS1 sequences, out of more than 6,000 present in the database, possessing this previously unrecognized tandem PL (TPL) domain (Table **S1**). The geographical distribution (based on the country of isolation as reported in the database) and the phylogenetic tree of these viruses (based on their NS1 protein sequences), are shown in Fig. **S1** and **S2**, respectively. As shown in Fig. **1 c**, only five versions of this domain, each differing from the others by single aminoacidic substitutions, appear to be present in this restricted subset of globally circulating AI viruses belonging to different H and N subtypes. The KRYMARRVESEV domain has been found in isolates throughout the world, while the KRYMERRVESEI is reported so far only for a cluster of closely related H7N2 viruses isolated in North America. The KRYMARRIESEV domain has been found in isolates from three geographical locations corresponding to three separate genetic clusters: Canada, comprising different H and N combinations; Chile, all belonging to the H7N3 genotype and Germany, with three isolates of the H10N7 genotype. These German isolates are also the oldest, dating back to 1949. The Chile isolates cluster genetically close to the H7N1 viruses of the italian epidemics analysed in this study, bearing the KRYMARRIESEV domain. The KRYMARRVESEI domain is present in 8 isolates falling in three separate genetic clusters. Five from China, of the H6N2, H1N1 and H9N2 genotypes, one from Canada, of the H3N8 genotype and two from US, of the H6N1 genotype. Interestingly, the five Chinese isolates appear to be genetically close to the oldest H10N7 German isolates with the KRYMARRIESEV domain. Finally, the KRFMARRVESEV domain is found only in two isolates from Japan. Overall, these data indicate that AI viruses carrying different versions of the TPL domain are circulating worldwide both in wild birds and domestic poultry.

### Identification of cellular ligands for the novel tandem PDZ-domain of avian influenza NS1

**Figure 2:**
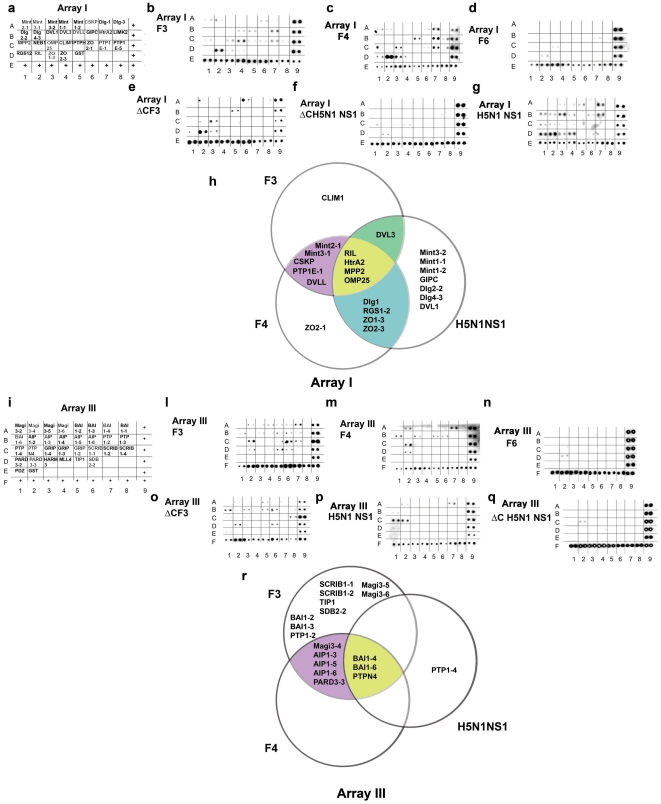
Interaction of avian influenza virus H7N1 NS1 proteins with cellular PDZ domains. **a**. Schematic representation of the different PDZ domains present on commercial PDZ Array I (Panomics). Each PDZ domain is present on the membrane in duplicate. Plus signs indicate positive controls for the anti-histidine antibody used for detection. **b**. Visualization of the interactions between the H7N1 full length NS1 protein (F3) with the PDZ domains on Array I. **c–g**. As in panel **b**, but with the NS1 F4_Δ225–230_, F6_Δ221–230_, ΔCF3_Δ225–230_, ΔCH5N1_Δ220–225_ and H5N1 NS1 proteins, respectively. **h**. Venn diagram of the interactions detected by the Array I. Violet: common interactions between F3 and F4_Δ225–230_ NS1 protein; green, common interactions between F3 and H5N1 NS1 proteins; cyan, common interactions between the F4_Δ225–230_ and H5N1 NS1 proteins; yellow, common interactions among the three NS1 proteins. **i** Schematic representation of the different PDZ domains present on commercial PDZ Array I (Panomics). Each PDZ domain is present on the membrane in duplicate. Plus signs indicate positive controls for the anti-histidine antibody used for detection. **l**. Visualization of the interactions between the H7N1 full length NS1 protein (F3) with the PDZ domains on Array I. **m–q**. As in panel **l**, but with the NS1 F4_Δ225–230_, F6_Δ221–230_, ΔCF3_Δ225–230_, ΔCH5N1_Δ220–225_ and H5N1 NS1 proteins, respectively. **r**. Venn diagram of the interactions detected by the Array I. Violet: common interactions between F3 and F4_Δ225–230_ NS1 protein; yellow, common interactions among the three NS1 proteins.

The PDZ binding properties of the novel TPL domain were tested on protein arrays (Figure 2), representing 58 different types of human PDZ domains (schematically drawn in Fig. **2 a** and **2 i**). As summarized in Fig. **2 h** and **2 r**, the full length NS1 protein F3 was able to bind 28 human cellular PDZ domains. The 6- aa naturally truncated NS1 F4_Δ225–230_, of the HPAI H7N1 viral strain, bearing only the PL domain II (Fig. **1 a**), interacted with 22 PDZ domains, but only 18 were in common with F3, suggesting that deletion of the first PL domain also changed the PDZ-domain binding specificity the protein. When an identical truncation was engineered into the full length F3 protein to obtain the mutant ΔCF3_Δ225–230_ (Fig. **1 a**) a pattern of interaction similar to F4_Δ225–230_, was observed (Fig. **2 e** and **2 o**), demonstrating that the differences between F3 and F4_Δ225–230_ NS1 proteins were not strain specific but dependent on the different PL domain arrangement. Finally, the naturally occurring 10-aa truncated NS1 protein F6_Δ221–230_, of the LPAI H7N1 with no PL domains (Fig. **1 a**), showed no interaction with the PDZ domains in both arrays (Fig. **2 d** and **2 n**), as expected. The F3, F4_Δ225–230_ and F6_Δ221–230_ NS1 proteins, although originating from different isolates, showed >99% identity at the aa level (Fig. **1 a**). In order to compare the PDZ binding characteristics of more distantly related AI strains, the NS1 protein of the HPAI H5N1 A/HK/213/03 isolate was tested. The H5N1 NS1 possesses a consensus PL domain I, but its PL domain II deviates from the consensus due to a single aa change from a hydrophobic to a positively charged residue (Fig. **1 a**). As shown in Fig. **2 h** and **2 r**, the H5N1 protein recognized a set of 20 PDZ domains, of which only 8 overlapped with the PDZ domains recognized by the F3 protein. Interestingly, the H5N1 and F4_Δ225–230_ NS1 proteins recognized 11 PDZ domains in common, four of which were specifically bound by these two proteins and not by F3. Deletion of the PL domain I of the H5N1 NS1 (protein ΔCH5N1_Δ220–225_), resulted in the complete loss of interaction with the PDZ domains (Fig. **2 f** and **2 q**), indicating that its PL domain II was not functional, as predicted by the sequence. Thus, PDZ-domain binding by AI NS1 proteins requires the presence of at least one functional C-ter PL domain.

**Figure 3:**
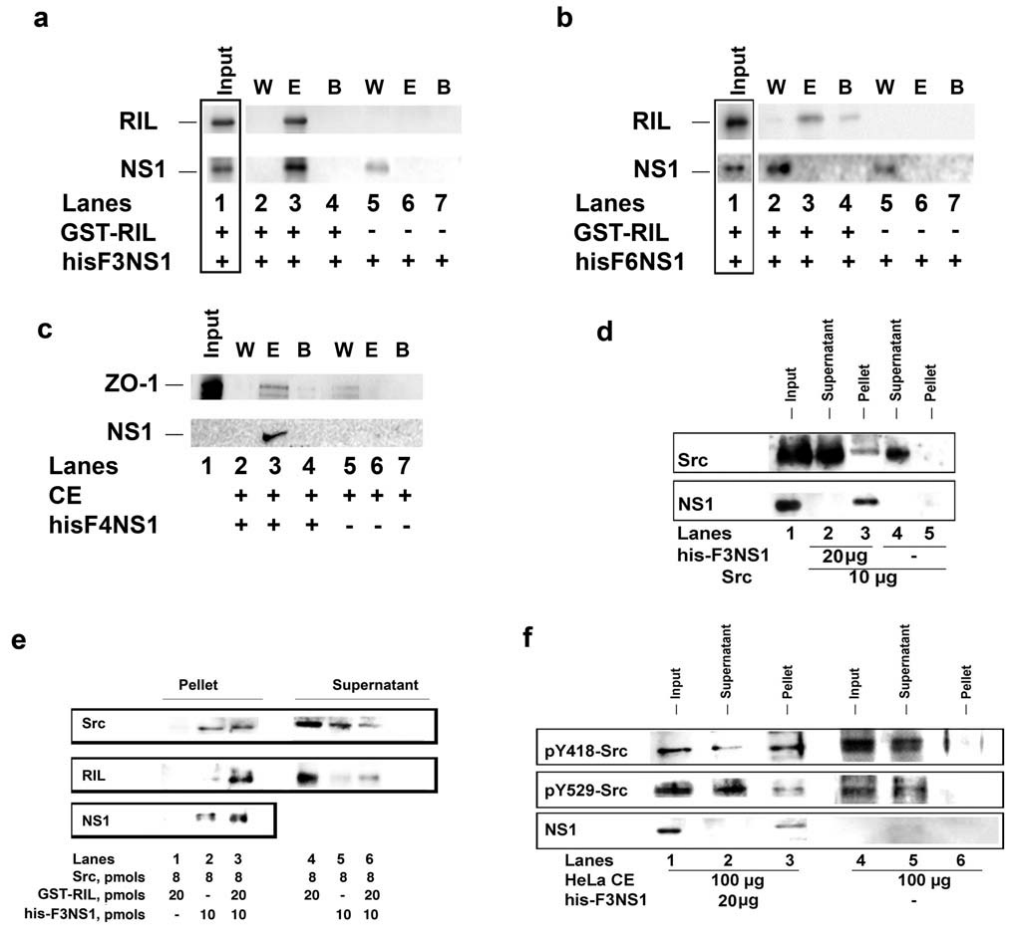
The H7N1 NS1 protein binds the cellular proteins RIL and Src. **a**. Pull-down analysis of the interaction of human RIL with the H7N1 F3 protein. 10 µg of recombinant his-tagged F3 NS1 was added to glutahione (GSH) sepharose beads in the presence (lanes 2–4) or in the absence (lanes 5–7) of 10 µg of recombinant human GST-tagged RIL. Beads were washed with PBS1×. Bound proteins were luted with 10 mM GSH, separated on SDS-PAGE and detected by western blot with anti-GST (RIL) or anti.his (NS1) antibodies. W, wash; E, elution; B, beads. Lane 1, control western blot with input proteins. **b**. As in panel a, but with the F4_Δ225–230_ protein. **c**. Pull-down of recombinant ZO1. Cell lysates overexpressing human ZO1 have been incubated with NiNTA beads in the presence (lanes 2–4) or in the absence (lanes 5–7) of recombinant his-tagged F4Δ225–230 protein. W, wash with 10 mM imidazole; E, elution with 500 mM imidazole; B, beads pellet. **d**. Recombinant GST-tagged human Src kinase was incubated in the presence (lanes 2,3) or in the absence (lanes 4, 5) of recombinant his-tagged H7N1 F3 NS1 protein. Proteins were pulled down with his-tag affinity NiNTA-agarose beads. Anti-Src or anti-his antibodies were used for the detection of Src and NS1, respectively. Lane 1, input proteins. Lanes 2 and 4, proteins detected in the supernatant in the presence or absence of F3, respectively. Lanes 3 and 5, pulled down proteins in the presence or absence of F3, respectively. **e**. Recombinant untagged human Src was added to NiNTA beads in the absence (lanes 1, 4) or in the presence of recombinant his-tagged F3NS1 (lanes 2, 3; 5, 6) and in the absence (lanes 2, 5) or in the presence of recombinant human GST-tagged RIL (lanes 3, 6). Lanes 1–3: pulled down proteins. Lanes 4–6, supernatants. Proteins were revealed by immunoblot with anti-Src (upper panel), RIL (middle panel) or his (bottom panel) antibodies. **f**. HeLa cell extracts were incubated in the presence (lanes 2, 3) or in the absence (lanes 5, 6) of his-tagged F3 NS1. Proteins were pulled down with his-tag affinity NiNTA-agarose beads. Lanes 2, 5:, proteins detected in the supernatant in the presence or absence of F3, respectively. Lanes 3, 6: pulled down proteins in the presence or absence of F3, respectively. Lanes 1, 4: inputs. Antibodies specific for the phosphorylated Y418 (pY418-Src) or Y529 phosphorylated (pY-529-Src) Src residues were used for the detection of the active and inactive Src forms, respectively. Anti-his antibodies were used for the detection of NS1.

Our array analysis identified the known NS1 interactors MAGI3 and Dlg1 [9]–[11] as binding to at least two of the three NS1 proteins analyzed (Fig. 2 **h** and **r**). Seven cellular PDZ domains, belonging to six different proteins, were also identified, which were able to bind both the H7N1 NS1 proteins and the H5N1 NS1, including the known NS1 interactor MAGI1 [11] (Table **S2**), Analysis of their PDZ domains allowed the identification of three blocks of high homology (Fig. **S3 a**), defining a PDZ-consensus for the interaction with AI NS1 proteins. The strongest signal from the arrays for the F3, F4_Δ225–230_, ΔCF3_Δ225–230_ and H5N1 NS1 proteins, was observed with the human Reversion-Induced LIM protein (RIL). To independently confirm that the interaction observed with the isolated PDZ domain of RIL on the arrays, reflected true protein-protein interactions, recombinant F4_Δ225–230_ and F6_Δ221–230_ proteins were used in pull-down assays with recombinant GST-RIL expressed in *E.coli* bound to GSH-sepharose beads. As shown in Fig. **3**, RIL interacted only with F4_Δ225–230_ (Fig. **3 a**) but not with F6_Δ221–230_ (Fig. **3 b**). In addition, one protein (ZO1) was identified in our array analysis as a specific interactor of the F4_Δ225–230_, ΔCF3_Δ225–230_ and H5N1 (Fig. **2**) NS1 proteins. Cell extracts containing the overexpressed recombinant full length ZO1 were then incubated with NiNTA-agarose beads in the absence or in the presence of recombinant his-tagged F4_Δ225–230_ NS1. As shown in Fig. **3 c**, ZO1 was eluted from the beads only in the presence of NS1 (lane 3), but not in its absence (lane 6). These results confirmed that the interactions with the isolated PDZ domains, detected by the arrays, could also be reproduced with intact full length proteins.

Sequences homologous to the PL domain I of NS1 were present in several PDZ-binding cellular proteins. A search of the human protein database with the consensus for the novel PL domain II of H7N1 NS1, returned 43 proteins bearing matching sequences at their extreme C-ter. Five of these proteins are reported in the database to interact with cellular PDZ proteins (Table **S3**). Among these cellular PDZ proteins, LIMK1 has been identified in a genome-wide RNA interference screening, as a host factor important for influenza virus replication [19]. In addition, the protein Dlg1 was identifed by our arrays and also independently shown to bind AI NS1 in published studies [9], [10]. The database query also reported the protein ZO1, which was identified by our array. Interestingly, a recent study showed that ZO1 was mislocalized during infection with AI viruses carrying the consensus -ESEV- PL motif (analogous to PLI), as a consequence of the interaction between NS1 and Dlg1 [11]. Thus, our data seem to suggest that AI NS1 can also directly interact with ZO1. Analysis of these five proteins and their respective ligands listed in Table **S3**, using the STRING interaction database [20], [21], revealed that six out of ten proteins were linked through experimentally confirmed physical interactions (Fig.**S3 b**). Interestingly, sequence alignment of the PDZ domains of the cellular interactors of the five proteins listed in Table **S3**, revealed significant homology with the PDZ domain of RIL (Table **S4**), which emerged as the strongest interactor of avian H7N1 NS1 proteins in our arrays. Collectively, the data of the arrays together with this bioinformatic analysis, suggest that the PL domain II, analogous to the one exposed by the 6- aa truncated H7N1 NS1, is also present in human proteins.

### The NS1 protein from H7N1 avian influenza virus physically interacts with both human RIL and Src proteins

RIL has been shown to interact with the active form of the tyrosine kinase Src, playing a major role in its downregulation [22]. Its interaction with NS1 might suggest an involvement of the viral protein in the RIL-Src axis. Notably, avian NS1 proteins possess two Src-homology type 3 binding (SHB) domains (Fig. **1 a**): SHB domain 1 which mediates the interaction of AI NS1 with the p85 beta subunit of the PI3K kinase [23], and SHB domain 2 which mediates the interaction with the proteins Crk/CrkL [24]. Interestingly, the SHB domain 2 (-PPLPPK-) of H7N1 NS1 is immediately upstream to the TPL (Fig. **1 a**). Thus, the ability of AI NS1 to bind Src was tested in pull down assays with recombinant human Src kinase. As shown in Fig. **3 e**, Src was retained on the Ni-NTA beads only in the presence of his-tagged F3 NS1 (compare lane 3 with lane 5). A molar excess of RIL did not disrupt the complex between NS1 and Src, so that a ternary complex was isolated in the absence of the peptide substrate (Fig. **3 f**, lane 3). Finally, his-tagged F3 NS1 protein was able to pull down endogenous Src from HeLa cells extracts (Fig. **3 g**, compare lane 3 with lane 6). Probing the pulled down Src (Fig. **3 g**, lane 3) and the input protein (Fig. **3 g**, lane 1), with antibodies specific for the active (pY418) or inactive (pY529) forms of Src, suggested that F3 preferentially associated with the active form of Src.

### The H7N1 NS1 proteins stimulate the human Src tyrosine kinase

**Figure 4:**
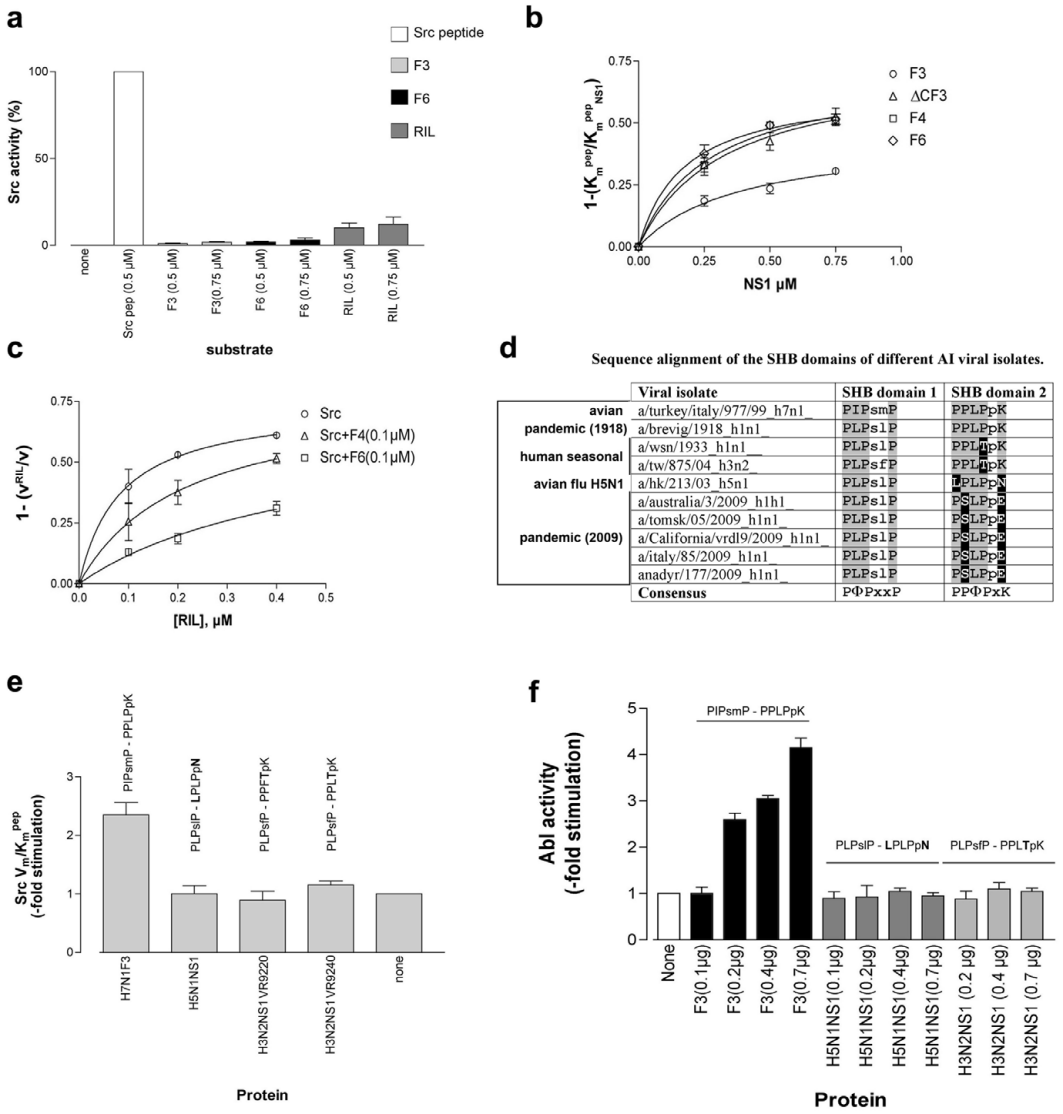
The H7N1 NS1 protein stimulates Src activity through its SH3-binding domain. **a**. *In vitro* Src kinase reactions were carried out in the absence or presence of the Src peptide (white bar) or increasing amounts of F3 (light grey bars), F6Δ221–230 (black bars) or human RIL proteins (dark grey bars) as substrates. Activity is expressed as relative to the reaction with the Src peptide substrate (100%). Values are the means of duplicate experiments. Error bars represent ±SD. **b**. Dose-dependent increase of the affinity of Src for its peptide substrate as a function of NS1 concentrations. Apparent peptide affinity values determined in the absence (K_m_^pep^) or in the presence (K_m_^pep^_NS1_) of NS1, were used to calculate the relative affinity increase (1-K_m_^pep^/K_m_^pep^_NS1_). Values are the means of calculations from three independent determinations of the affinity constants. Error bars are ±SD. **c**. Stimulation of Src activity, expressed as the increase in the ratio of enzyme velocity in the presence (v^RIL^) and in the absence (v) of increasing amounts of RIL alone (circles) or in the presence of fixed doses of F4_Δ225–230_ (triangles), or F6_Δ221–230_ (squares) NS1 proteins. Values are the means of three independent replicates. Error bars are ±SD. **d**. Sequence alignment of the SHB domains 1 and 2 of different influenza viruses. Conserved residues with respect to the consensus are shaded in grey, non-synonimous substitutions in conserved residues are in boxed in black. **e**. Stimulation of Src catalytic efficiency (V_m_/K_m_^pep^) by H7N1 F3 NS1, H5N1 NS1, or the H3N2 2007 epidemic human isolates VR9220 and VR9240 NS1. Each bar represents the mean value of three independent determinations of the V_m_/K_m_^pep^. Error bars are ± S.D. **f**. *In vitro* Abl kinase reactions were performed in the presence of the specific Abl peptide substrate and in the absence (white bars) or in the presence of increasing amounts of F3 (grey bars), H5N1 NS1 (black bars) or human influenza H3N2 NS1 (light rey bars) proteins. Experiments were in triplicate. Error bars represent ±SD.

The previously unrecognized interaction between NS1 and Src, prompted us to investigate its functional significance. H7N1 NS1 proteins were not efficient substrates for *in vitro* Src kinase reactions (Fig. **4 a**), but were able to increase the peptide substrate utilization efficiency (V_max_/K_m_^pep^ ratios) of Src (Table 1). The F4_Δ225–230_ and ΔCF3_Δ225–230_ proteins, bearing PL domain II only, or the F6_Δ221–230_, bearing no PL domains, were more efficient in stimulating Src (5.5-, 3.1- and 4.8- fold increase of V_max_/K_m_^pep^, respectively, Table 1) than the full length F3 protein with the intact TPL (2- fold increase, Table 1). Accordingly, the apparent affinities of the viral NS1 proteins for Src were higher (lower absolute K_d_^NS1^ values) for the F4_Δ225–230_, the ΔCF3_Δ225–230_ and the F6_Δ221–230_ proteins (Table 1 and Fig. **4 b**). Collectively, these data show, for the first time, that NS1 can stimulate human Src and that this ability can be influenced by the PL domain arrangement.

**Table 1:** Kinetic constants for the interaction of Src with its peptide substrate and the NS1 and RIL proteins.

Protein	K_d_^NS1^ (µM)	K_m_^pep^ (µM)	V_max_ (pmol×min^−1^)	V_m_/K_m_^pep^ (pmol×min^−1^×µM^−1^)	K_d_^RIL^ (µM)
none		106(±14)	7(±0.5)	0.07	0.08±0.02
F3(H7N1)	0.4(±0.05)	62(±5)	8(±0.5)	0.13	n.d.
F4(H7N1)_Δ225–230_	0.2(±0.04)	25(±3)	9.9(±0.7)	0.39	0.23±0.05
ΔCF3(H7N1)_Δ225–230_	0.25(±0.06)	40(±5)	9(±1)	0.22	n.d.
F6(H7N1)_Δ221–230_	0.15(±0.04)	23(±3)	7.8(±0.7)	0.34	0.47±0.08
RIL		71(±4)	8(±1)	0.11	n.a.

n.d., not determined; n.a., not applicable.

RIL alone was not a substrate for Src (Fig. **4 a**) nor did it significantly stimulate Src activity (Table 1). When increasing amounts of RIL were titrated in the kinase reaction, in the absence or presence of increasing fixed doses of either F4_Δ225–230_, or F6_Δ221–230_ proteins (Fig. **4 c**), the apparent affinity of Src for RIL was decreased (increase in the absolute K_d_^RIL^ values, Table 1) by the presence of the NS1 proteins, suggesting that, in the presence of the peptide and ATP substrates, the binding of NS1 and RIL to Src was mutually competitive.

### The SH3-binding domain 2 of H7N1 NS1 proteins is essential for the interaction with Src

RIL has been shown to interact with both the kinase and SH2 domains of Src [22]. We hypothesized that NS1 might bind, instead, to the SH3 domain of Src through one of its SHB domains. The general consensus sequences for the AI SHB domains 1 and 2 are -PØPxxP- and –PPØPxK/R-, respectively, where Ø indicates hydrophobic aminoacids. Sequence analysis (Fig. **4 d**) showed that the SHB domain 1 is generally conserved among influenza viruses, while the SHB domain 2 is more variable. The -PPLPPK- sequence is present in the 1918 H1N1 “Spanish flu” virus (A/Brevig_Mission/1918), while the most recent H1N1v 2009 pandemic virus, as well as the human H1N1 and H3N2 seasonal strains, show different aa substitutions, leading to the loss of conserved residues. The H5N1 NS1 protein carries aminoacid substitutions in its SHB domain 2 leading to the loss of two conserved residues (-LPLPPN-) with respect to the consensus, while all the H7N1 NS1 proteins retain a perfect match (-PPLPPK-). We cloned the NS1 protein from two seasonal H3N2 human influenza isolates of the 2007 epidemic (VR9220 and VR9240), bearing an intact SHB domain 1, but with one aminoacid substitution in a conserved Pro of SHB domain 2 (-PPØTPK-), an compared them to the F3 and H5N1 NS1 protein for their ability to stimulate Src. No stimulation of Src was observed by the full length H5N1 NS1, or by the two H3N2 NS1 proteins (Fig. **4 e**), suggesting that an intact SHB domain 2 was required for the interaction.

### The SH3-binding domain 2 of H7N1 NS1 proteins is a general interaction module for cellular tyrosine kinases

Since the SHB domain is a general protein module for interaction with SH3-containing tyrosine kinases, we tested whether AI NS1 could also bind other Src-unrelated tyrosine kinases, as an indication of SHB domain functionality. Indeed, H7N1 NS1 was able to stimulate the *in vitro* activity of the human tyrosine kinase c-Abl (Fig. **4 f**). When similar experiments were carried out with the full length H5N1 NS, or the two H3N2 human influenza NS1 proteins, no stimulation of the activity of Abl (Fig. **4 f**), was observed, further confirming the importance of an intact -PPØPxK/R- consensus SHB domain 2 for this interaction. The SHB domain 2 sequence -PPLPPK- was present in 326/427 (76.3%) of the AI viral isolates identified in this study bearing the TPL domain. Alignment of the different SHB domain 2 sequences found in all the 427 AI isolates, to the consensus -PPØPxK- sequence (Table **S1**), indicated a degree of conservation between 93% and 100% for all the essential positions, with the exception of the Pro in fourth position, which showed a non-conservative substitutions with a Ser in 16% of the isolates (Table **S1**). This residue has been previously recognized as a host marker in pandemic viruses [25]. It is possible that the different degree of conservation in the SHB domain 2 among mammalian and AI viruses, reflects different selective pressures.

### The activation state of cellular Src during infection is differently modulated by H7N1 viruses expressing the full length or the truncated NS1 protein

**Figure 5:**
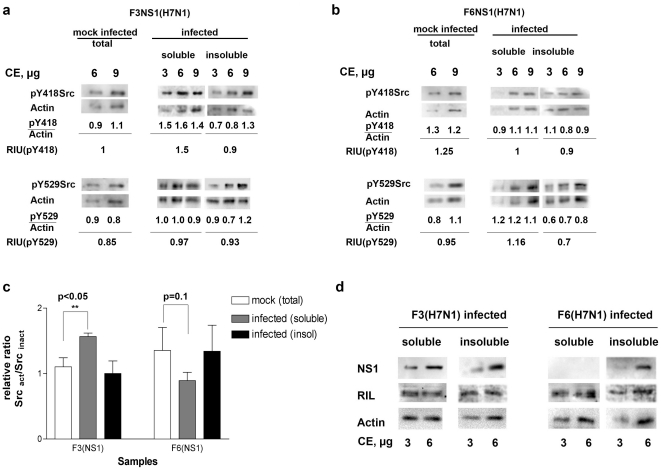
The activation state of cellular Src during infection is differently modulated by H7N1 viruses expressing the full length or the truncated NS1 protein. a. A representative Western blot analysis of the active (Y418 phosphorylated) or inactive (Y529 phosphorylated) forms of Src in cells infected with the with natural 977 H7N1 strain expressing full length NS1. Intensities normalized to the actin controls are indicated below each corresponding lane. Mean values for the intensities normalized to actin are indicated below each set as relative intensity units (RIU). b. as in panel a, but for cells infected with the 4426 virus expressing the F6?221-230 NS1 protein. c. Normalized RIU values for each form, were used to derive the relative ratios for active vs. inactive Src. Values are the means derived from the western blots of two independent infection experiments, as exemplified in panels a and b. Each western blot has been loaded with two (mocked) or three (infected) different amounts of crude extract, in order to obtain replicated measurements for each infection experiment. Error bars are ±S.D. d. Western blot analysis of NS1 and RIL proteins in the cell extracts used for the experiments shown in panels a and b.

Endogenous Src is present as an active or inactive protein in the cell, depending on its phosphorylation status [26], [27]. A precise balance of the different phosphorylated forms is essential for cellular homeostasis. The cellular protein RIL has been shown to be important for the maintenance of the active vs. inactive Src equilbrium [22]. The physical and functional interaction of the H7N1 NS1 proteins with both RIL and Src, may suggest that the activation state of Src within the cell during the infection might be influenced by the ability of H7N1 NS1 proteins to interact with RIL. To start addressing this question, the phosphorylation status of endogenous Src was then assessed, through western blotting analysis with antibodies specific for the active (pY418) or inactive (pY529) forms of Src, in A549 (human alveolar basal epithelial cancer) cells infected with the r977 or the r4426 recombinant viral strains (Fig. 5 **a**, **b**). These viruses contain the full length F3 (r977) and the truncated F6_Δ221–230_ (r4226) proteins, respectively, with an A/turkey/Italy/977/99 H7N1 backbone (see also Fig. **1 a**). Intensities of each band were normalized to the intensity of actin revealed on the same membrane. The resulting relative intensity units (RIU) were used to calculate the Src active/Src inactive ratios, which are shown in Fig. **5 c**). The virus expressing full length F3 NS1 protein, caused a statistically significant increase (p<0.05) of the active form of Src in the soluble fraction, while the virus expressing the truncated F6_Δ221–230_ protein did not significantly change the balance between active and inactive Src. In addition, while the full length F3 NS1 was detected in both the soluble and insoluble fractions, the F6_Δ221–230_ protein was selectively retained in the insoluble fraction only (Fig. **5 d**), suggesting that the 10- aa deletion of F6_Δ221–230_ NS1 changed the intracellular distribution of the protein in the infected cells. This is consistent with the observation that C-ter truncations in H7N1 NS1 protein cause its recruitment to the nucleolar compartment [28]. No changes in the intracellular distribution of RIL were observed (Fig. **5 d**). Overall, these result indicate that the truncation occurring in the F6_Δ221–230_ NS1 protein, while not affecting its ability to interact and activate Src kinase in vitro, prevents Src activation in the infected cells, suggesting an important role of the PL domain in mediating NS1 association with Src, in vivo, presumably through interaction with the Src regulator RIL.

## Discussion

Interaction of AI NS1 with the PDZ domains of cellular proteins has been shown to influence viral pathogenicity. H5N1 NS1 interacts with the cellular PDZ proteins Scribble and Dlg, altering the JAK/STAT signalling pathway and protecting infected cells from apoptosis [9]–[11]. However, the avian PL sequence (-ESEV-) seems to affect viral pathogenesis in a strain- and host-dependent manner [13], [14]. For example, while reintroduction of the fully functional 1918 H1N1 PL domain in the NS1 protein of the human H3N2 A/WSN/33 virus, caused enhanced pathogenicity in mice [12], the same modification of the 2009 H1N1v pandemic virus did not result in enhanced replication or transmissibility, suggesting that other factors also contribute to viral pathogenicity [29]. Comparison of the NS1 sequences of human circulating H1N1 and H3N2 with the new H1N1v pandemic strain, showed that the last 50–60 aa at the C-ter of NS1 were the less conserved, with aa identity ranging from 41.7 to 67.2%, with respect to the 78–84% aa identity showed at the level of the entire protein [30]. This suggests that the extreme C-ter domain (including the PL domain), is subjected to selective pressure during circulation in novel hosts. Significant host-specific evolution of the PL domain of swine influenza viruses have also been suggested by detailed sequence comparison among strains circulating in different geographical areas in the last >30 years, indicating host-specific positive selection at the level of the PL domain, as a result of cross-species transmission [31]. Indeed, human, equine and swine influenza strains, including the 2009 H1N1v pandemic virus, do not harbour a PL domain, suggesting that loss of PDZ-binding capability may be one of the steps involved in adaptation of avian viruses to new hosts [32].

Our phylogenetic analysis of the Italian H7N1 isolates (Fig. **S4**), further corroborates this view. We showed that HPAI viruses circulating in the mid-phase of the epidemic with the F4_Δ225–230_ protein, possessing a PL domain II only, arose from LPAI viruses with full length NS1 F3, harbouring an intact TPL domain. On the contrary, the LPAI viruses circulating at the end of the epidemics with the NS1 mutant F6_Δ221–230_, bearing a complete loss of PL domains, originated from a separate viral pool, suggesting that the 10-aa deletion of F6_Δ221–230_ NS1, conferred a selective advantage over the circulating viruses with the 6-aa deleted F4_Δ225–230_ NS1.

Due to its potential binding to multiple partners, NS1 can interfere with different cellular pathways on a competition basis, with some interactions being more stable or more probable than others. In particular, the AI virus NS1 protein activates the cellular PI3K kinase by disrupting the interaction between the catalytic p110 subunit and its negative regulatory subunit p85, through physical binding to the p110 kinase domain [23].

Here, we showed that AI virus NS1 can interfere with the Src-RIL regulatory axis in a manner which is influenced by the PL arrangement. RIL was shown to bind to the phosphatase PTPL1, allowing its association with the active form of Src [22]. PTPL1 then catalyzes the dephosphorylation of the regulatory residue Y418 of Src, resulting in kinase inactivation. Our array analysis showed that full length H7N1 and H5N1 NS1 proteins can interact through their PL domains with both RIL and PTPL1, potentially being able to disrupt their interaction. The H7N1 NS1 proteins were also found in this study to bind and stimulate human Src kinase through their SHB domain 2, while the H5N1 NS1 protein was not. We showed that loss of PL domain I (as in the F4_Δ225–230_ protein) abolishes interaction with PTPL1 but not with RIL and Src. Deletion of the entire TPL (as in the F6_Δ221–230_ mutant) from the H7N1 NS1, abolishes the interaction with RIL, but still allows interaction of the viral protein with Src. Thus, the H7N1 NS1 protein seems to carry the potential for a wider range of interactions than its H5N1 counterpart, thanks to its TPL and SHB domain 2.

We propose a model, whereby AI NS1 replaces PTPL1 as the interacting partner of RIL, preventing Src inactivation. RIL binds to the SH2 and kinase domain of Src, allowing NS1 interaction with the SH3 domain. Binding of a protein substrate displaces RIL from the Src-NS1-RIL complex, whereby NS1 increases the catalytic efficiency of Src. Loss of the PL domain prevents NS1 interaction with RIL. Interestingly, the F6_Δ221–230_ mutant lacking all PL domains, was found to be sequestered into the insoluble fraction, upon lysis of infected cells, while the full length F3 NS1 protein was also retained in the soluble part, along with RIL and Src. The soluble fraction of active Src was indeed specifically enriched in infected cells expressing full length NS1, but not the F6_Δ221–230_ mutant. Src subcellular localization is subjected to complex regulation and Src has been found associated not only to the cytoplasmic side of the plasma membrane, but also to the endoplasmatic reticulum, Golgi apparatus and cytoskeletal components [33]. Thus, it is possible that NS1 requires interaction with RIL to be relocalized at specific cellular compartments together with Src. Src transduces signals to many cellular pathways, affecting cell proliferation, cell motility, apoptosis, immune signalling [34], [35]. The exact role of Src activation in the AI virus replication cycle is still unknown, however, a recent report showed that IL-1 dependent inflammatory response due to influenza A viral infection, was dependent on Src activation and was completely abrogated by PP2 treatment [36]. Thus, it is tempting to speculate that viruses bearing NS1 proteins able to upregulate Src activity through the RIL axis, might be expected to induce a stronger inflammatory response than viruses with NS1 proteins lacking RIL- or Src-binding capabilities. We have shown that NS1 interaction with Src required the SHB domain 2. Interestingly, human circulating H1N1 and H3N2 strains as well as the 2009 pandemic H1N1v, all have mutations in the SHB domain 2, which prevent interaction with Src, suggesting again a possible host-specific adaptation mechanism.

If loss or inactivation by mutation of the PL domain is a step involved in adaptation of AI viruses to new hosts, those strains bearing two PL domains in tandem (TPL), as those reported here, would present an higher genetic barrier to adaptation. The comparison of the PDZ-binding specificity of the F3 and F4 H7N1 NS1 proteins, indicated that the set of PDZ domains bound by the NS1 protein bearing both TPL I and II (F3), was significantly different from the one recognized by the NS1 protein bearing TPL II only (F4). This suggests that these two domains are not identical and that deletion of the TPL I can alter the network of interactions between AI NS1 and cellular PDZ proteins, possibly affecting pathogenesis. Thus, AI viruses bearing a TPL may represent a potential threat in case of cross-species infections [37]. Interestingly, by sequence alignment, we have found the novel TPL domain arrangement also in a group of H5N1 isolates circulating in China (A/Env/HK/437/99, acc. no. Q9EA66, Q9EA72, Q77NF2, Q9DGY0), antigenically and genetically related to the H5N1 virus responsible for the human AI outbreak of 1997 [38]. Thus, the potential interaction of their NS1 proteins with essential human cellular pathways warrants further investigation.

A therapeutic strategy that can reduce the emergence of viral resistance towards antiviral drugs is the selective targeting of host factors required for viral replication. We have recently provided a successful example of such an approach towards HIV-1 infection [39]. In this study, by analyzing the occurrence of AI-like PL domains in human PDZ-binding proteins, a restricted number of proteins potentially mediating host-pathogen relationships in AI virus replication have been identified. These findings might be further expanded to identify novel anti-influenza targets and drugs.

## Materials and Methods

### Chemicals

Labelled [gamma-^32^P]- ATP was from GE Healthcare. All other chemical reagents were from Merck and Fluka.

### Enzymes and proteins

Baculovirus-produced recombinant purified active human Src and Abl were purchased from Upstate (Lake Placid, NY).

### Cloning of NS1 proteins and construction of deletion mutants

The genes of NS1 proteins used in this study were: NS1 wild type (225 aa) from avian influenza virus A/HK/213/03 H5N1, NS1 wild type (230 aa) from A/Turkey/Italy/977/99 H7N1 (F3), NS1 containing the deletion of 225–230 aa from A/Turkey/Italy/4580/99 H7N1 (F4_Δ225–230_), NS1 containing the deletion of 220–230 aa from A/Turkey/Italy/4426/00 H7N1 (F6_Δ221–230_). The *ns1* genes were amplified using a standard RT-PCR protocol and cloned into the pQE30 expression vector. For detailed cloning procedures see the Supplementary Information.

### Reverse genetics

The recombinant viruses r977 and r4426 expressing the full length F3 and the truncated F6_Δ221–230_ NS1 respectively were generated in an A/Turkey/Italy/977/1999 genetic background as described [14].

### Protein purification

The expression of His_6_-NS1 proteins was induced in BL21 (DE3) cells transformed with the expression vector pQE30-NS1 as described in Supplementary Information. Soluble His_6_-tagged NS1 proteins were purified by chromatography through an FPLC-Ni-NTA column as described (see Supplementary Information). Recombinant GST-RIL was purified from the pGEX-5x-1-RIL expressing vector (15) by gluthathione-conjugated GSH-sepharose beads (GE Healthcare) as described in Supplementary Information.

### Pull down assay

Recombinant GST-tagged RIL (10 µg) was bound to GSH-sepharose beads. His-tagged recombinant F3 or F4 proteins (10 µg) were added in PBS1× and incubated at 4°C O/N. Bound proteins were recovered by centrifugation, eluted with 10 mM glutathione and resolved by SDS-PAGE, followed by Western blotting with anti-His_6_ and anti-GST IgG antibodies.

Recombinant His-tagged F3 (20 µg) coupled to NiNTA-agarose beads in PBS 1× were mixed with 10 µg of recombinant GST-tagged human Src kinase and incubated at 4°C O/N. Bound proteins were recovered by centrifugation and resolved by SDS-PAGE, followed by Western blotting with anti-His_6_ and anti-Src rabbit IgG antibody (for detailed protocol see Supplementary Information).

Alternatively, recombinant His-tagged F3 (20 µg) coupled to NiNTA-agarose beads in PBS 1× were mixed with HeLa cell lysate for 1 h at 4°C. Pulled down proteins were resolved on SDS-PAGE 10% gels. Samples were analyzed by Western blotting using either primary rabbit anti-Src [pY^418^] (to detect the active form of Src kinase) or anti-Src [pY^529^] (to detect the inactive form of Src kinase) antibodies (Biosource). For detailed protocol see Supplementary Information.

### PDZ domain arrays

PDZ domain arrays representative of 58 human PDZ domains (Array I and III, Panomics) were used. Each membrane was incubated with the manufacturer's Wash Buffer for 30 min and then blocked with the manufacturer's Blocking Buffer 1×, and shaken at room temperature for 2 h. After two washes with Wash Buffer 1×, the membranes were placed on a shaker and incubated with 15 µg/ml of each NS1 proteins, diluted in Blocking Buffer 1×, for 2 h at room temperature. After incubation, the membranes were washed three times with Wash Buffer 1×, and incubated with Anti-Histidine-HRP-conjugated for 2 h at room temperature. For identification of the spots detection solution was prepared according to the manufacturer's specifications.

### Kinase Assays

Src activity was measured in a filter-binding assay using a commercial kit (Src Assay Kit, Upstate), according to the manufacturer's protocol, using the specific Src peptide substrate [KVEKIGEGTYGVVYK] and in the presence of 0.012 µM of Src and 0.160 pmol of [γ-^32^P]ATP. Unlabelled ATP was added to reach the final concentrations as indicated in the figure legends. Abl activity was measured in a filter-binding assay using an Abl specific peptide substrate (Abtide, Upstate), 0.012 µM [γ-^32^P]ATP, 0.022 µM c-Abl. Reaction conditions were (in a final volume of 10 µl): 25 mM Tris-HCl pH 7.5, 1 mM DTT. Unlabelled ATP/Mg^++^ (1∶1 M/M) mix was added to reach the final ATP concentration of 100 µM. Reactions were incubated for 10 min at 30°C. Samples (9 µl) were spotted on paper cellulose filters which were washed according to the manufacturer's protocol. Filter-bound radioactivity was measured by liquid scintillation with a Microbeta-Trilux apparatus (Perkin-Elmer).

### Viral infection

For the western blot experiments, 1×10^5^ human alveolar basal epithelial cancer cells (A549, ATCC no. CCL-185) were infected following standard protocols for 48 hrs at an M.O.I. of 1 with either recombinant virus r977 or r4426 with the addition of 1.5 µg/ml of TPCK trypsin.

### Cell lysis

A549 cells were harvested following infection and incubated on ice for 30 min in 50 mM Tris-HCl pH 8.0, 0.1% SDS, 0.35 M NaCl, 0.25% Triton in the presence of protease inhibitor cocktail. Cells were then centrifuged 10 min at 4°C (20000×g). The supernatant was kept as the soluble fraction. The pellet was extracted by sonication (3×5 s) in 0.5 M NaCl, supplemented with 1×Laemli Buffer, heated 5 min at 100°C, centrifuged (30 min 20000×g) and the supernatant kept as the insoluble fraction.

### Kinetic Analysis

The kinetic constants were derived by fitting the experimental data to the appropriate rate equations, as detailed in supplementary Information. Curves were obtained by non-linear least squares computer fitting of the data to the equations with the program GraphPad Prism 3.0.

All sequence alignments were generated with the tool CLUSTALW (http://www.ebi.ac.uk/Tools/clustalw2/). Similarity searches were performed with the tools BLAST (http://www.expasy.ch/tools/blast/) and PROSITE (http://www.expasy.ch/tools/scanprosite/). Network interaction analysis was performed by STRING (http://string-db.org/).

## Supporting Information

Figure S1 **Geographical distribution of th AI isolates bearing the novel TPL domain.** The map is based on the locations of the reported identification of the different isolates present in the database. The different versions of the TPL domain are indicated with different colours. Due to sampling bias, the frequency of each variant in the database does not necessarily correlate with the actual natural distribution of the different viruses.
(PDF)

Figure S2 **Phylogenetic tree of the AI viral isolates bearing the novel TPL domain.** The tree was generated starting from the aminoacidic sequences of 195/427 isolates present in the database, including all the sequences of the KRYMERRVESEI, KRYMARRIESEV and KRYMARRVESEI groups and 97/325 sequences of the most common KRYMARRVESEV group. Isolates with the same TPL domain version are grouped by coloured frames. The tree was generated by the platform Phylogeny.fr (http://www.phylogeny.fr/) with the program sMUSCLE, Gblocks and PhyML (Dereeper A., et al. Nucl. Acids Res. 2008, 36, Web server issue W45-9). Tree was drawn by the program TreeDyn.
(PDF)

Figure S3 **Analysis of cellular PL type II domains.** Sequence alignment of the human cellular PDZ-domains interacting with H7N1 and H5N1 NS1 proteins, identified by protein arrays analysis. Blocks of identical residues (in red) are highlighted in yellow. Similar aminoacids are highlighted in green. The derived consensus is shown (in red) at the bottom of the panel. Ø, hydrophobic residues; ¥, aliphatic residues. **b**. STRING interaction network of the cellular proteins bearing a PL domain type-II (KIF13B, CADM1, CNTAP-2) and their respective PDZ-ligands (DLG1, MPP3, CASK). Colour coded connecting lines as per STRING nomenclature are as follows: purple, experimentally proven physical interactions; green, co-cited in the same article (PubMed); cyan, linked through functional homology. **c** Expanded STRING interaction network built from the cellular proteins (circled in red) with PL domain II (KIF13B, CNTAP-2, CADM1) and their respective PDZ ligands (DLG1, CASK, MPP3). The thickness of the connecting lines is proportional to the STRING score (likelihood of interaction).
(PDF)

Figure S4 **Phylogenetic tree of the NS1 proteins from H7N1 AI viruses isolated during the Italian 1999–2001 epidemic.** The tree was generated using the neighbour-joining method as implemented by the MEGA4 program (Tamura K. et al. (2007), Mol. Biol. Evol. 24, 1596–1599). Green circles identify isolates with a full length (230 aa) protein; yellow triangles identify isolates with a 6 aa C-ter truncation (Δ225–230); red squares identify isolates with a 10 aa C-ter truncation (Δ221–230).
(PDF)

Table S1 **TPL an SHB2 domains of AI viruses grouped according to the corresponding hemagglutinin (H) subtypes.**
(PDF)

Table S2 **Cellular PDZ proteins interacting with H7N1 and H5N1 NS1 proteins.**
(PDF)

Table S3 **PDZ-binding human proteins bearing NS1 PL II homologous domains and their cellular ligands.**
(PDF)

Table S4 **Alignment of human PDZ domains interacting with cellular PL domain II-homologs, to the PDZ domain of the human protein RIL, interacting with AI NS1.**
(PDF)
