# Supplementary figures and images for: The PDZ-Ligand and Src-Homology Type 3 Domains of Epidemic Avian Influenza Virus NS1 Protein Modulate Human Src Kinase Activity during Viral Infection
Source: PLoS One. 2011 Nov 14;6(11):e27789. doi: 10.1371/journal.pone.0027789 (PMC3215730; doi:10.1371/journal.pone.0027789)

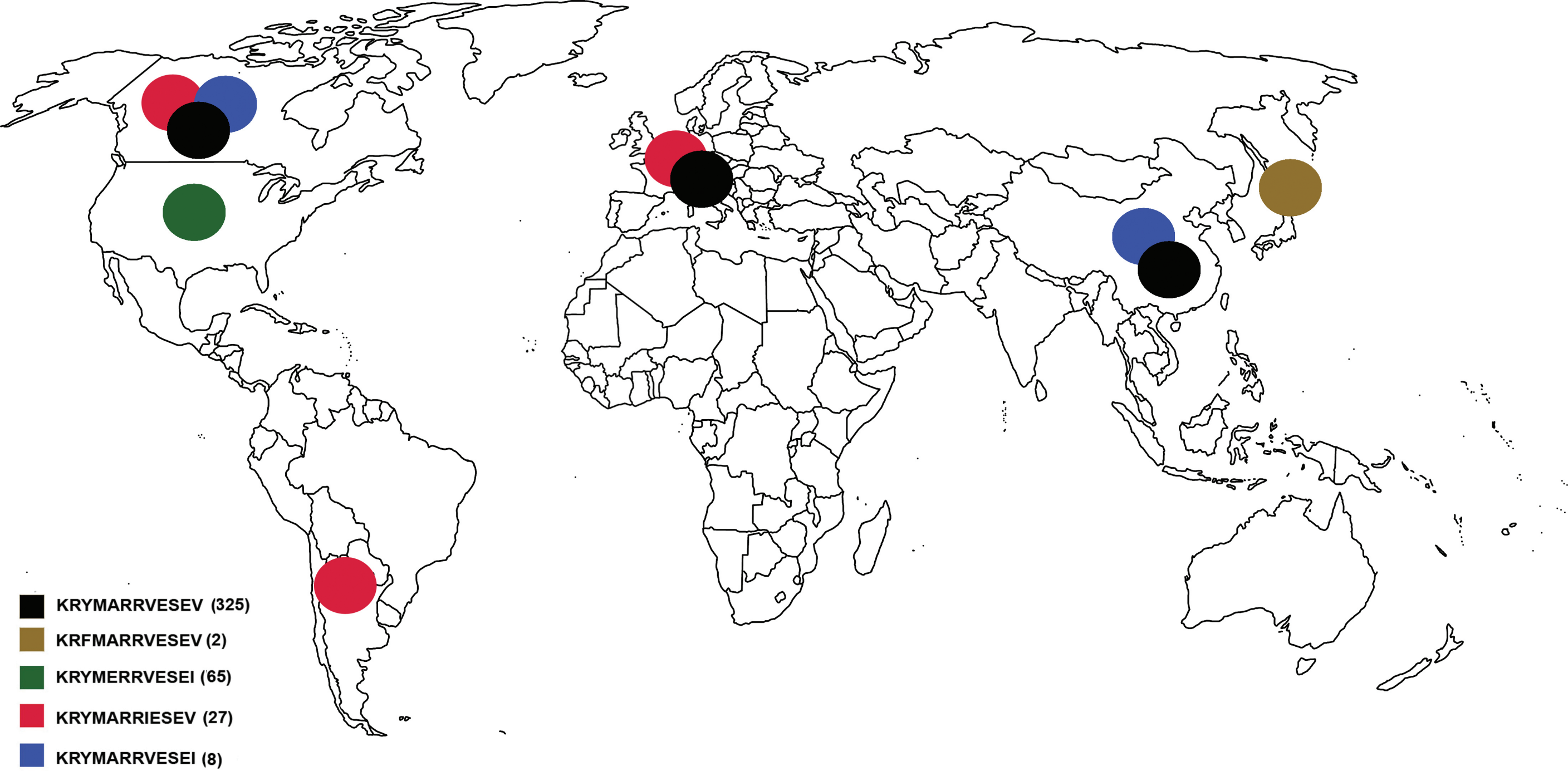

Figure S1

Supplement: Figure S1 — Geographical distribution of th AI isolates bearing the novel TPL domain. The map is based on the locations of the reported identification of the different isolates present in the database. The different versions of the TPL domain are indicated with different colours. Due to sampling bias, the frequency of each variant in the database does not necessarily correlate with the actual natural distribution of the different viruses. (PDF) [file pone.0027789.s001.pdf]

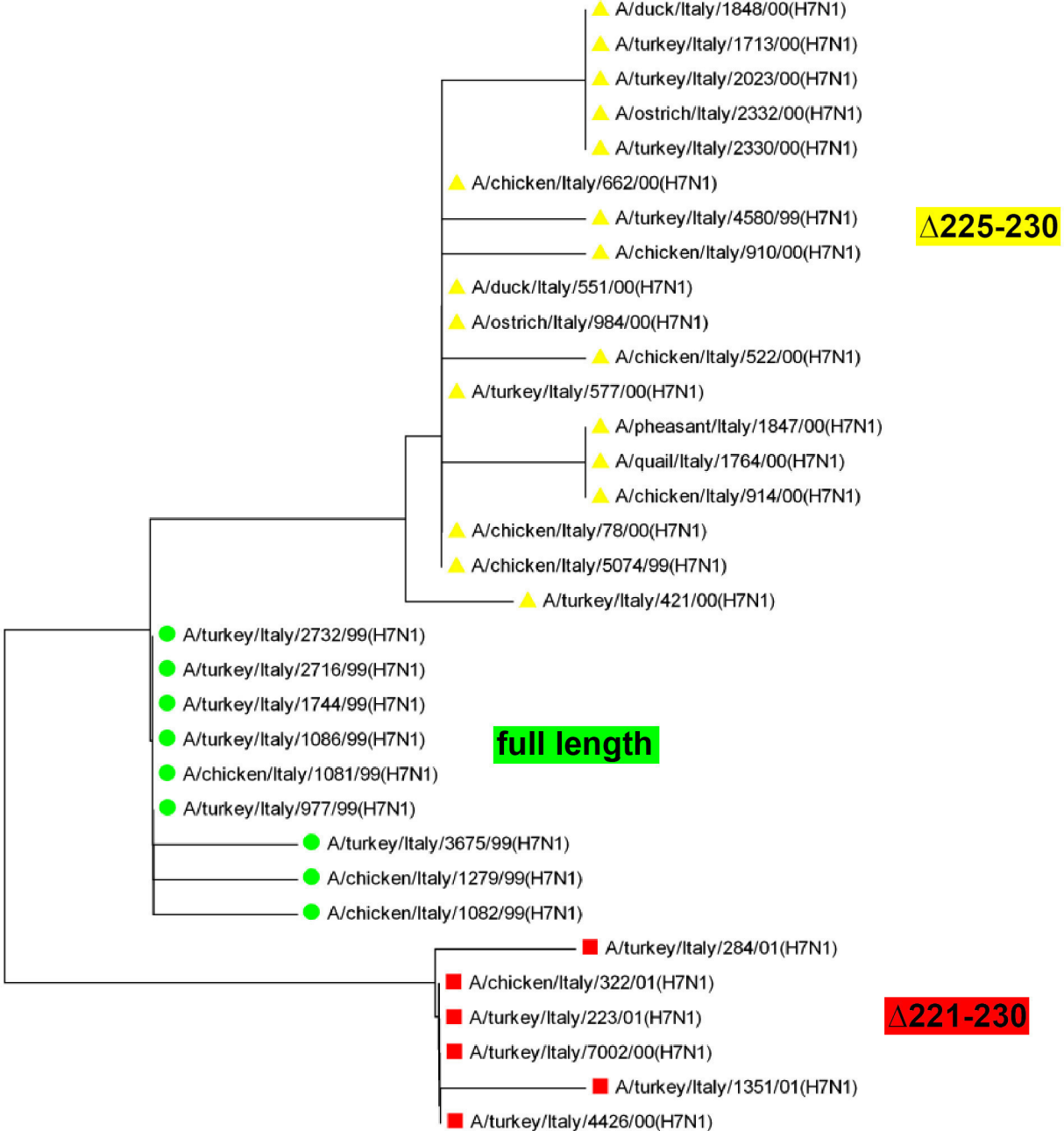

Supplement: Figure S4 — Phylogenetic tree of the NS1 proteins from H7N1 AI viruses isolated during the Italian 1999–2001 epidemic. The tree was generated using the neighbour-joining method as implemented by the MEGA4 program (Tamura K. et al. (2007), Mol. Biol. Evol. 24, 1596–1599). Green circles identify isolates with a full length (230 aa) protein; yellow triangles identify isolates with a 6 aa C-ter truncation (Δ225–230); red squares identify isolates with a 10 aa C-ter truncation (Δ221–230). (PDF) [file pone.0027789.s004.pdf]
