# Supplementary material for: The PDZ-Ligand and Src-Homology Type 3 Domains of Epidemic Avian Influenza Virus NS1 Protein Modulate Human Src Kinase Activity during Viral Infection
Source: PLoS One. 2011 Nov 14;6(11):e27789. doi: 10.1371/journal.pone.0027789 (PMC3215730; doi:10.1371/journal.pone.0027789)
Supplement: Figure S3 — Analysis of cellular PL type II domains. Sequence alignment of the human cellular PDZ-domains interacting with H7N1 and H5N1 NS1 proteins, identified by protein arrays analysis. Blocks of identical residues (in red) are highlighted in yellow. Similar aminoacids are highlighted in green. The derived consensus is shown (in red) at the bottom of the panel. Ø, hydrophobic residues; ¥, aliphatic residues. b. STRING interaction network of the cellular proteins bearing a PL domain type-II (KIF13B, CADM1, CNTAP-2) and their respective PDZ-ligands (DLG1, MPP3, CASK). Colour coded connecting lines as per STRING nomenclature are as follows: purple, experimentally proven physical interactions; green, co-cited in the same article (PubMed); cyan, linked through functional homology. c Expanded STRING interaction network built from the cellular proteins (circled in red) with PL domain II (KIF13B, CNTAP-2, CADM1) and their respective PDZ ligands (DLG1, CASK, MPP3). The thickness of the connecting lines is proportional to the STRING score (likelihood of interaction). (PDF) [file pone.0027789.s003.pdf]

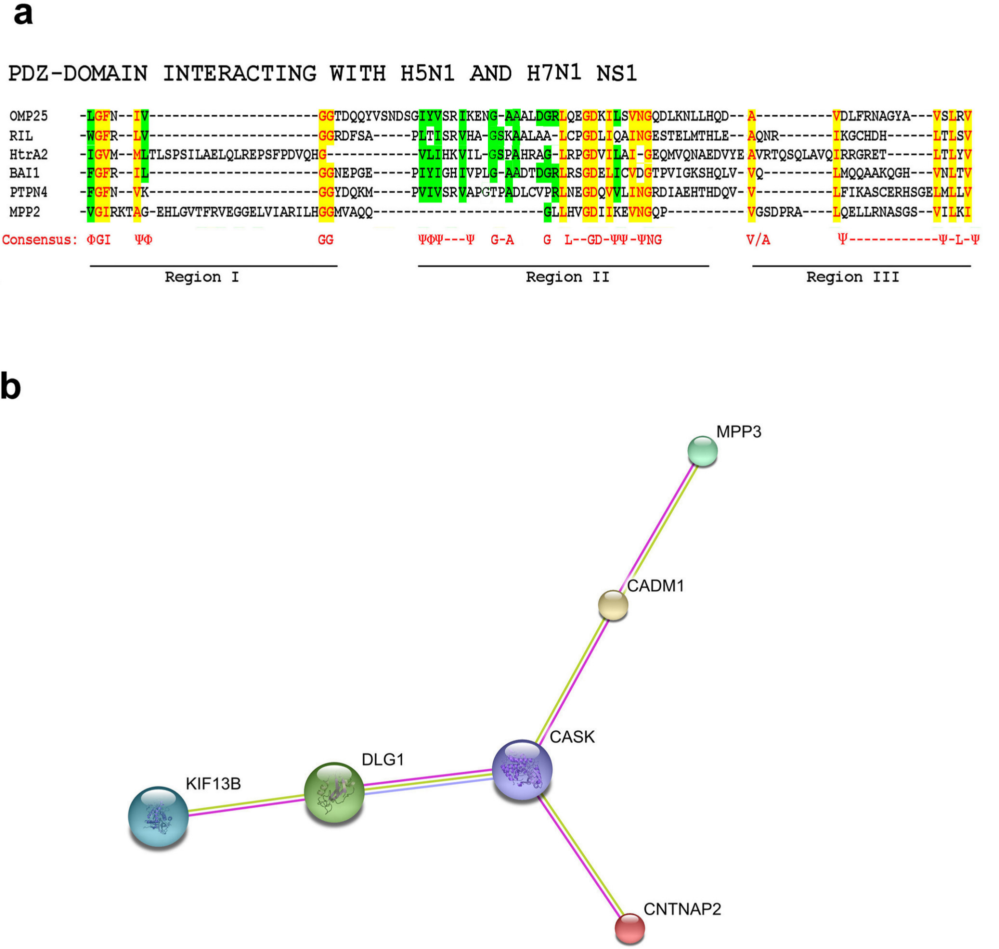
a
PDZ-DOMAIN INTERACTING WITH H5N1 AND H7N1 NS1
OMP25 -LGFN--IV-----------------------GGTDQQYVSNDSGIYVSRIKENG-AAALDGRLQEGDKILSVNGQDLKNLLHQD--A----------VDLFRNAGYA---VSLRV
RIL -WGFR--LV-----------------------GGRDFSA-----PLTISRVHA-GSKAALAA-LCPGDLIQAINGESTELMTHLE--AQNR--------IKGCHDH------LTLSV
HtrA2 -IGVM--MLTLSPSILAELQLREPSFPDVQHG-------------VLIHKVIL-GSPAHRAG-LRPGDVILAI-GEQMVQNAEDVYE AVRTQSQLAVQIRRGRET------LTLYV
BAI1 -FGFR--IL-----------------------GGNEPGE-----PIYIGHIVPLG-AADTDGRLRSGDELICVDGTPVIGKSHQLV-VQ---------LMQQAAKQGH---VNLTV
PTPN4 -FGFN--VK-----------------------GGYDQKM-----PVIVSRVAPGTPADLCVPRLNEGDQVVLINGRDIAEHTHDQV-V-----------LFIKASCERHSGELMLLV
MPP2 -VGIRKTAG-EHLGVTFRVEGGELVIARILHGGMVAQQ------------------------GLLHVGDIIKEVNGQP----------VGSDPRA----LQELLRNASGS--VILKI
Consensus: ΦGI ΨΦ GG ΨΦΨ---Ψ G-A G L--GD-ΨΨ-ΨNG V/A Ψ-----------Ψ-L-Ψ
Region I
Region II
Region III
b
MPP3
CADM1
CASK
DLG1
KIF13B
CNTNAP2
