# Supplementary material for: The PDZ-Ligand and Src-Homology Type 3 Domains of Epidemic Avian Influenza Virus NS1 Protein Modulate Human Src Kinase Activity during Viral Infection
Source: PLoS One. 2011 Nov 14;6(11):e27789. doi: 10.1371/journal.pone.0027789 (PMC3215730; doi:10.1371/journal.pone.0027789)
Supplement: Table S1 — TPL an SHB2 domains of AI viruses grouped according to the corresponding hemagglutinin (H) subtypes. (PDF) [file pone.0027789.s005.pdf]

Table S1. TPL and SHB2 domains of AI viruses grouped according to the corresponding hemagglutinin (H) subtypes.

	Total:427<br><br>TPL Sequence	Total:427<br><br>SHB2 Sequence
Consensus	KRΦMARRΨESEΨ	PPϕPxK
	KRYMARRVESEV (325)	PPLPpK (289/325)<br><br>PSLPpK (27/325)<br><br>PSFPpK (4/325)<br><br>PPLSpK (1/325)<br><br>LPLPpK (1/325)<br><br>PPLPsK (1/325)<br><br>PPFPpK (3/325)
	KRYMERRVESEI (65)	PPLSpK (49/65)<br><br>LPLSpK (2/65)<br><br>LPISpK (1/65)<br><br>PPLStK (12/65)<br><br>PPLSsK (1/65)
	KRYMARRIESEV (27)	PPLPpK (27/27)
	KRYMARRVESEI (8)	PPLPpK (8/8)
	KRFMARRVESEV (2)	PPLPpK (2/2)
Genotypes	TPL Sequence	
H1N1	KRYMARRVESEI	
H3N6/8	KRYMARRIESEV	
H4N5/6/8/9	KRYMARRIESEV<br><br>KRFMARRVESEV	

H5N1/2/3	KRYMARRIESEV<br>KRYMARRVESEV	
H6N1/2/3	KRYMARRVESEI<br>KRYMARRIESEV<br>KRFMARRVESEV	
H7N1/2/3	KRYMARRVESEV<br>KRYMERRVESEI<br>KRYMARRIESEV	
H9N2	KRYMARRVESEI	
H10N7	KRYMARRIESEV	
H11N9	KRYMARRIESEV	

Φ = hydrophobic; Ψ = aliphatic
