# Supplementary material for: The PDZ-Ligand and Src-Homology Type 3 Domains of Epidemic Avian Influenza Virus NS1 Protein Modulate Human Src Kinase Activity during Viral Infection
Source: PLoS One. 2011 Nov 14;6(11):e27789. doi: 10.1371/journal.pone.0027789 (PMC3215730; doi:10.1371/journal.pone.0027789)
Supplement: Table S2 — Cellular PDZ proteins interacting with H7N1 and H5N1 NS1 proteins. (PDF) [file pone.0027789.s006.pdf]

Table S2. Cellular PDZ proteins interacting with H7N1 and H5N1 NS1 proteins

Acc. Number (UniProtKB)	Names	Functions
P57105	OMP25, SYNJ2BP	Mitochondrial membrane protein. Binds synaptojanin-2 binding protein.
O43464	HTRA2	Mitochondrial membrane serine protease. Involved in apoptosis.
Q96QZ7	BAIP1, MAGI1	Membrane associated guanylate kinase. Scaffolding protein at cell-cell junctions.
P29074	PTPN4	Protein tyrosine phosphatase. Involved in signal transduction.
Q14168	MPP2, DLG2	Belongs to MAGUK family. Involved in signal transduction.
P50479	RIL, PDLIM4	Interacts with c-Src and actin. Tumor suppressor gene.
