# Supplementary material for: The PDZ-Ligand and Src-Homology Type 3 Domains of Epidemic Avian Influenza Virus NS1 Protein Modulate Human Src Kinase Activity during Viral Infection
Source: PLoS One. 2011 Nov 14;6(11):e27789. doi: 10.1371/journal.pone.0027789 (PMC3215730; doi:10.1371/journal.pone.0027789)
Supplement: Table S3 — PDZ-binding human proteins bearing NS1 PL II homologous domains and their cellular ligands. (PDF) [file pone.0027789.s007.pdf]

Table S3. PDZ-binding human proteins bearing NS1 PL II homologous domains and their cellular ligands

		PL domain (C-ter 6 aa)							
	position	-5	-4	-3	-2	-1	0		
**Protein**	**Acc. no. (UniProt)**							**PDZ Interactor**	**Acc. no. (UniProt)**
**LATS1**	O95835	R	D	L	V	Y	V	**LIMK1**	P53667
**CNTAP_2**	Q9UHC6	K	K	E	W	L	I	**CASK**	O14936
**CADM1**	Q9BY67	K	K	E	Y	F	I	**MPP3**	Q13368
**CXD3**	Q8N144	R	R	D	L	A	I	**ZO-1**	Q07157
**KIF13B**	Q9NQT8	R	K	S	W	A	S	**DLG1**	Q12959
**F4H7N1**		K	R	Y	M	A	R	**RIL**	P50479
**LATS1 match**		+	-	Φ	Φ	Φ	Φ		
**CNTP_2 match**		+	+	-	Φ	Φ	Φ		
**CADM1 match**		+	+	-	Φ	Φ	Φ		
**GJD3 match**		+	+	-	Φ	Φ	Φ		
**KIF13B**		+	+	*n*	Φ	Φ	*n*		
**F4H7N1 match**		+	+	Φ	Φ	Φ	+		

Legend: Φ = hydrophobic; Ψ = aliphatic, + = positively charged; - = negatively charged; *n* = neutral
