# Supplementary material for: The PDZ-Ligand and Src-Homology Type 3 Domains of Epidemic Avian Influenza Virus NS1 Protein Modulate Human Src Kinase Activity during Viral Infection
Source: PLoS One. 2011 Nov 14;6(11):e27789. doi: 10.1371/journal.pone.0027789 (PMC3215730; doi:10.1371/journal.pone.0027789)
Supplement: Table S4 — Alignment of human PDZ domains interacting with cellular PL domain II-homologs, to the PDZ domain of the human protein RIL, interacting with AI NS1. (PDF) [file pone.0027789.s008.pdf]

Table S4. Alignment of human PDZ domains interacting with cellular PL domain II-homologs, to the PDZ domain of the human protein RIL, interacting with AI NS1.

Protein	Acc. no. (UniProt)	PDZ domain
**LIMK1**	P53667	GLSVSIDPPHGPPG-------CGTEHSHTVRVQGVDPGCMSPDVKNSIHVGDRILEINGTPIRNVPLDEIDLLIQETSRLLQLTLEH
**CASK**	O14936	GITLKMNELNHC-IVARIMH-GGMIHRQGT-----------------LHVGDEIREINGISVANQTV-EQLQKMLREMRGS-ITFKI
**MPP3**	Q13368	GATIRRDEHSGAVVVARIMR-GGAADRSGL-----------------VHVGDELREVNGIAVLHKRPDEISQILAQSQGS--ITLKI
**ZO-1**	Q07157	GLRLASHIFVKEISQDSLAARDGN-----------------------IQEGDVVLKINGTVTENMSLTDAKTLIERSKGK--LKMVV
**DLG1**	Q12959	GLGFNIV--------------GGEDGEGIFISFILAGGPADLSGE--LRKGDRIISVNSVDLRAASH-EQAAAALKNAGQA-VTIVA
**RIL**	P50479	GFRLV----------------GGRDFSAPLTI-SVHAGSKAALAA--LCPGDLIQAINGESTELMTHLEAQNRIKGCHDH--LTLSV
