# Supplementary material for: The PDZ-Ligand and Src-Homology Type 3 Domains of Epidemic Avian Influenza Virus NS1 Protein Modulate Human Src Kinase Activity during Viral Infection
Source: PLoS One. 2011 Nov 14;6(11):e27789. doi: 10.1371/journal.pone.0027789 (PMC3215730; doi:10.1371/journal.pone.0027789)
Supplement: Figure S2 — Phylogenetic tree of the AI viral isolates bearing the novel TPL domain. The tree was generated starting from the aminoacidic sequences of 195/427 isolates present in the database, including all the sequences of the KRYMERRVESEI, KRYMARRIESEV and KRYMARRVESEI groups and 97/325 sequences of the most common KRYMARRVESEV group. Isolates with the same TPL domain version are grouped by coloured frames. The tree was generated by the platform Phylogeny.fr (http://www.phylogeny.fr/) with the program sMUSCLE, Gblocks and PhyML (Dereeper A., et al. Nucl. Acids Res. 2008, 36, Web server issue W45-9). Tree was drawn by the program TreeDyn. (PDF) [file pone.0027789.s002.pdf]

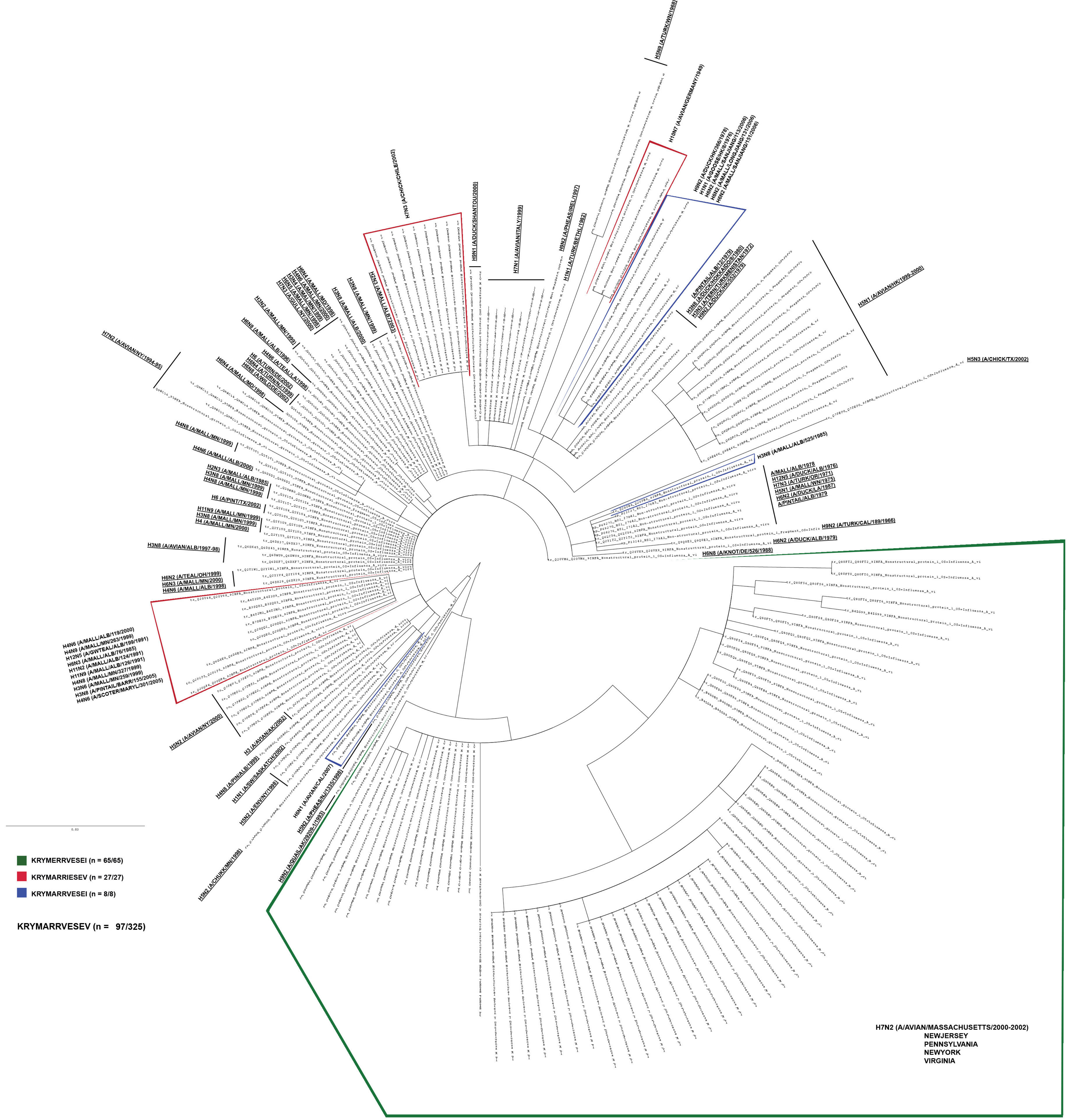
H5N3 (A/CHICK/TX/2002)
H3N8 (A/MALL/ALB/525/1985)
A/MALL/ALB/1978
H12N5 (A/DUCK/ALB/1976)
H7N3 (A/TURK/OR/1971)
H5N1 (A/MALL/WN/1975)
H6N2 (A/DUCK/LA/1987)
A/PINTAIL/ALB/1979
H9N2 (A/TURK/CAL/189/1966)
H6N2 (A/DUCK/ALB/1979)
H6N8 (A/KNOT/DE/526/1988)
H4N8 (A/MALL/MN/1999)
H4N6 (A/MALL/ALB/2000)
H2N3 (A/MALL/ALB/1985)
H3N8 (A/MALL/MN/1999)
H4N8 (A/MALL/MN/1999)
H6 (A/PINT/TX/2002)
H11N9 (A/MALL/MN/1999)
H3N8 (A/MALL/MN/1999)
H4 (A/MALL/MN/2000)
H3N8 (A/AVIAN/ALB/1997-98)
H6N2 (A/TEAL/OH/1999)
H6N3 (A/MALL/MN/2000)
H4N6 (A/MALL/ALB/1998)
H4N6 (A/MALL/ALB/119/2000)
H4N9 (A/MALL/MN/263/1999)
H12N5 (A/GWTEAL/ALB/199/1991)
H6N3 (A/MALL/ALB/76/1985)
H11N2 (A/MALL/ALB/124/1991)
H11N9 (A/MALL/ALB/126/1991)
H4N8 (A/MALL/MN/327/1999)
H3N6 (A/MALL/MN/259/1999)
H3N8 (A/PINTAIL/BARR/155/2005)
H4N6 (A/SCOTER/MARYL/301/2005)
H7N2 (A/AVIAN/MASSACHUSETTS/2000-2002)
NEWJERSEY
PENNSYLVANIA
NEWYORK
VIRGINIA
0.02
KRYMERRVESEI (n = 65/65)
KRYMARRIESEV (n = 27/27)
KRYMARRVESEI (n = 8/8)
KRYMARRVESEV (n = 97/325)

Figure S2.
